# Plasmacytoma in the Maxillary Jaw: A Diagnostic and Therapeutic Challenge

**DOI:** 10.3390/hematolrep16010003

**Published:** 2024-01-04

**Authors:** Sara Bernardi, Serena Bianchi, Ettore Lupi, Davide Gerardi, Guido Macchiarelli, Giuseppe Varvara

**Affiliations:** 1 Department of Life, Health and Environmental Sciences, University of L’Aquila, 67100 L’Aquila, Italy; serena.bianchi@univaq.it (S.B.); or davide.gerardi@studenti.unich.it (D.G.); guido.macchiarelli@univaq.it (G.M.); 2 Department of Biotechnological and Applied Clinical Sciences, University of L’Aquila, 67100 L’Aquila, Italy; ettore.lupi@graduate.univaq.it; 3 Department of Innovative Technologies in Medicine & Dentistry, University “G. d’Annunzio” of Chieti-Pescara, 66100 Chieti, Italy; gvarvara@unich.it

**Keywords:** plasmacytoma, oral health, upper jaw

## Abstract

Plasmacytoma is a neoplastic disorder originating from plasma cells, with bone and soft tissue being common sites of manifestation. This report presents the clinical and radiological findings of a 65-year-old female patient who presented with an exophytic lesion in the upper right lateral incisor region. The lesion appeared as a unilocular radiotransparent area in imaging tests. Following an excisional biopsy, histological and immunohistochemical evaluations confirmed the presence of mature plasmacellular elements and small infiltrates of B and T lymphocytes. The patient did not exhibit systemic manifestations of multiple myeloma. Surgical intervention, in the form of enucleation of the lesion combined with root canal treatment and apicoectomy, was performed. This case underscores the rare occurrence of plasmacytoma in the jaw region and highlights the importance of surgical management in cases where structural damage or functional impairment is present. Further research on novel treatment approaches is also mentioned, including targeted therapies, immunomodulatory agents, and monoclonal antibodies. The patient is currently under the care of a hematologist for further investigation and the choice of the most appropriate therapy.

## 1. Introduction

Plasmacytoma is a neoplastic disorder that arises from plasma cells, which are the final stage of B-lymphocyte maturation [1]. The most common locations for plasmacytoma are bone tissue and soft tissue, where clonal plasma cells produce monoclonal immunoglobulins, contributing to the growth of the tumor mass [2]. Plasmacytoma can be localized in the bone marrow as an isolated form, classified as “Solitary plasmacytoma” [3]. A rare area for the occurrence of plasmacytoma is in the head and neck region, particularly in the jaw area. The growth of plasmacytoma in the bone marrow leads to clinical and radiological manifestations such as bone pain, fractures, local soft tissue involvement resulting in swelling, and radiographic abnormalities [4].

When the presence of solitary plasmacytoma diffuses in the body systems, manifesting as the presence of light chains in the serum and/or urine, kidney damage, altered blood calcium levels (hypo- or hypercalcemia), anemia, leukopenia, and thrombocytopenia, the diagnosis may confirm the presence of multiple myeloma [4]. Solitary plasmacytoma has a better prognosis than multiple myeloma, and local recurrences after treatment are infrequent. However, the progression to multiple myeloma after five years from treatment is relatively rare [5].

Treatment approaches for plasmacytoma, whether in its solitary form or associated with multiple myeloma, involve various combinations such as local surgery, local irradiation, or chemotherapy [6]. Sensitive tests (M PROTEIN, free light chain (FLC), imaging, and Positron-Emitting Tomography–Computer Tomography (PET/CT)) are valuable for differentiating between the isolated presence of clonal plasma cells in the bone marrow and systemic manifestations of the disease. These tests help determine the long-term prognosis of solitary plasmacytoma and the risk of progression to multiple myeloma. Surgical treatment has shown positive outcomes in long-term prognosis, while irradiation and systemic therapy may not be recommended as standard treatment protocols [7]. Here, we present a rare case of the presence of a plasmacytoma in the upper jaw. The case is reported using the Case Report (CARE) Guidelines for reporting case reports [8]. 

## 2. The Case

A 65-year-old female patient came to our attention due to an exophytic lesion in correspondence with the upper right lateral incisor. The patient’s medical history did not report any positivity for relevant disease. 

**Figure 1:**
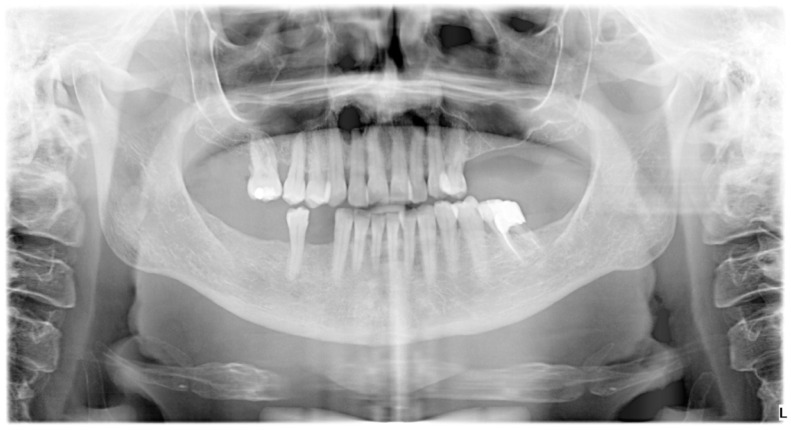
Orthopantomogram (OPG) showing a solitary, hollow area that appears dark on an X-ray image, in the front upper jaw on the right side, specifically around the base of the upper right lateral incisor.

**Figure 2:**
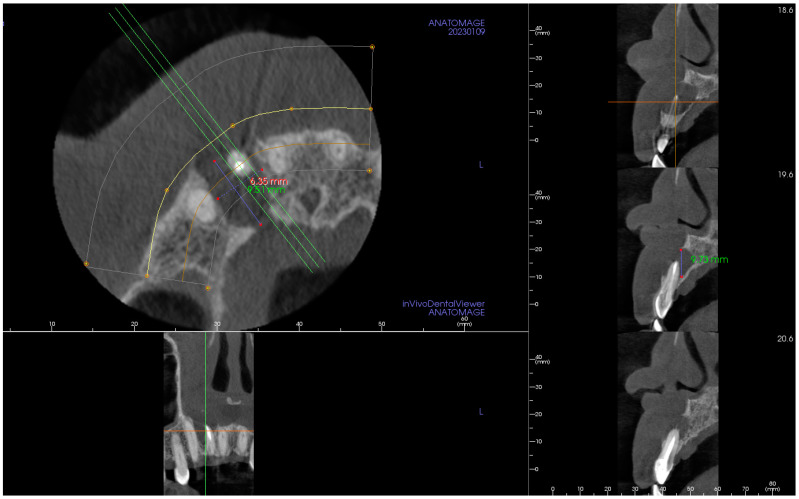
Cone-Beam Computed Tomography showing a three-dimensional image of the lesion, measuring 9.51 × 6.35 × 9.73 mm, on Panorex, Cross-Section, and Paraxial cuts, in correspondence with the upper right lateral incisor, after root canal treatment.

**Figure 3:**
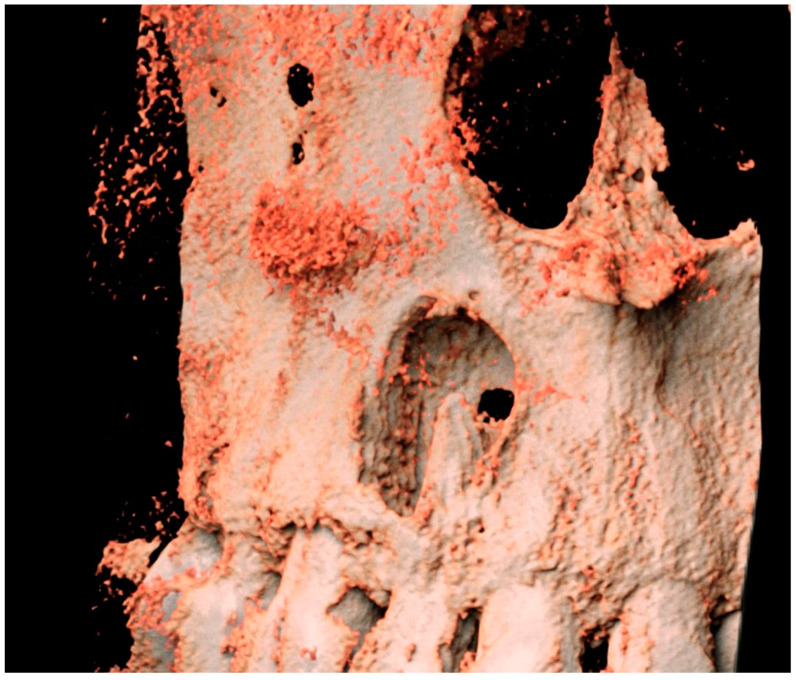
Three-dimensional volume rendering of the lesion obtained using Anatomage^®^ version 5.1.

**Figure 4:**
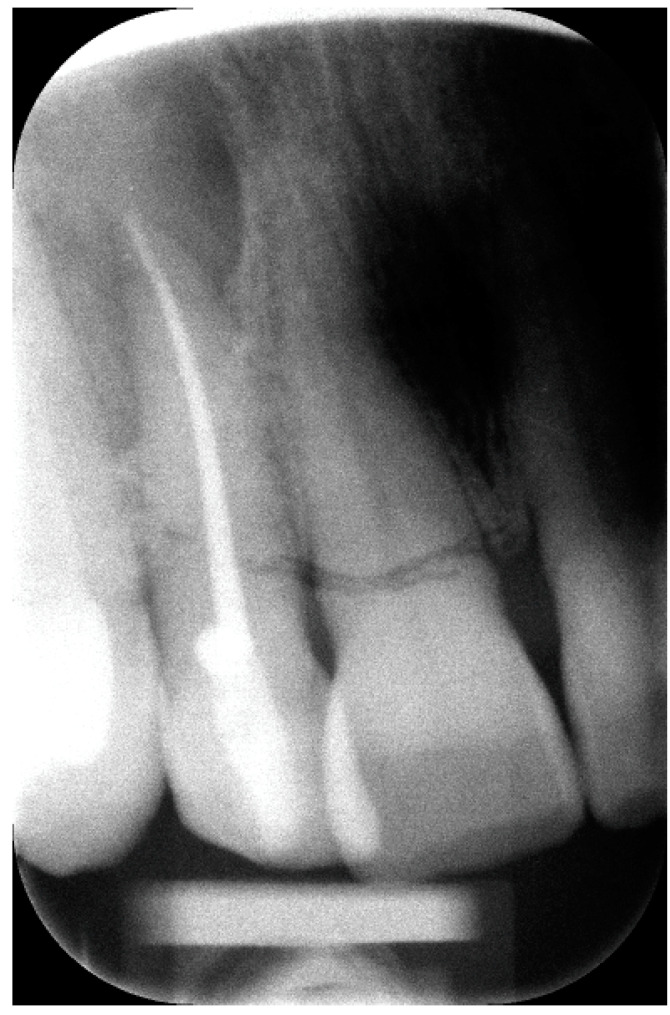
Intraoral Rx showing the lesion around the apical part of the apex of the upper right lateral incisor, after root canal treatment.

An orthopantomogram (OPG) (Figure 1), Cone-Beam Computed Tomography (CBCT) and a related 3D volume rendering (Figure 2 and Figure 3), as well as an intraoral radiograph (rx) (Figure 4) showed a single unilocular radiotransparent lesion in the right part of the anterior maxilla, in correspondence with the root of the upper right lateral incisor. 

**Figure 5:**
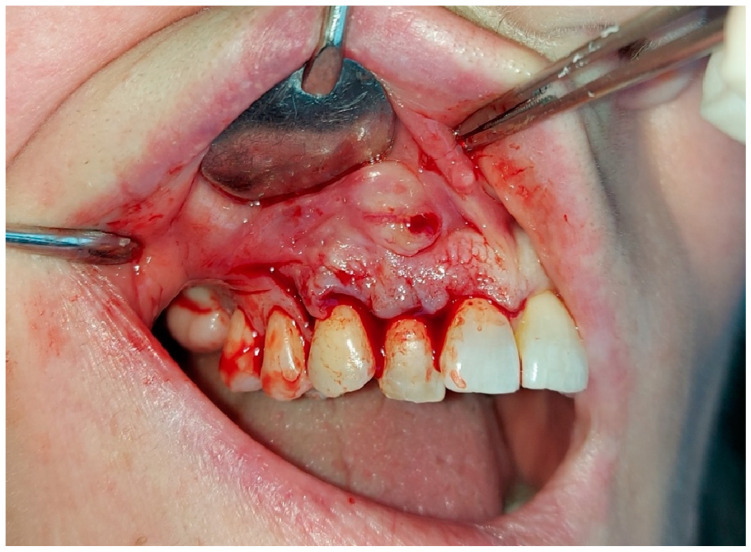
Exophytic lesion of the alveolar mucosa, with a diameter of one centimeter, without involving the keratinized gingiva.

The initial clinical examination indicated the presence of an exophytic lesion of the alveolar mucosa, with a diameter of one centimeter, without involving the keratinized gingiva (Figure 5), which was not associated with any symptoms. The upper right lateral incisor, which was involved in the lesion, responded positively to the vitality test. However, the patient underwent a root canal of the upper right lateral incisor as first treatment; then, after one week, an excisional biopsy was performed.

**Figure 6:**
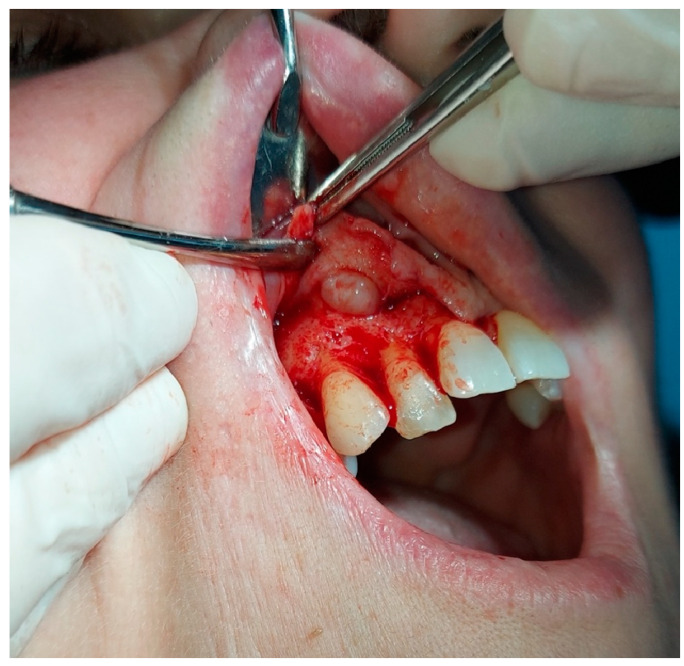
Capsulated lesion, not adherent to the surrounding tissues; the cortical bone which surrounded the lesion appeared cribriform.

**Figure 7:**
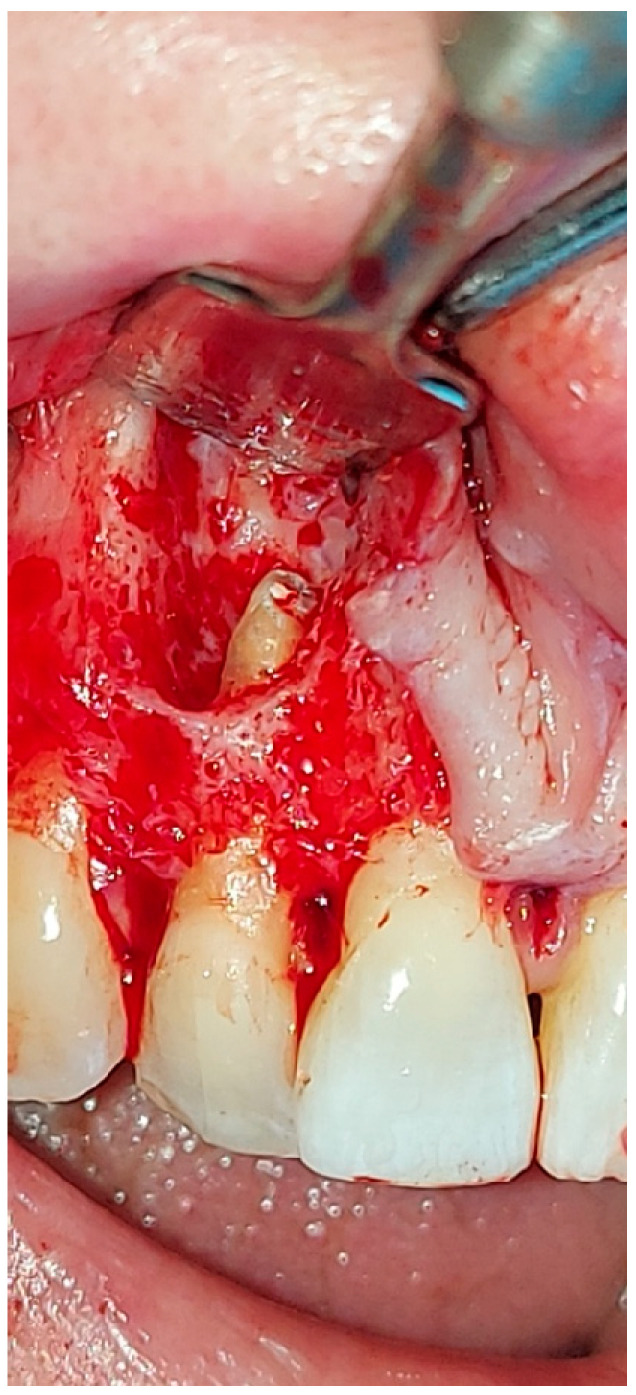
Lateral incisor after performing treatment of apicoectomy with mineral trioxide aggregate (MTA) applications.

Surgical inspection after the local surgery of the excisional biopsy showed a cortical bone surrounding the lesion, which appeared cribriform (Figure 6 and Figure 7).

**Figure 8:**
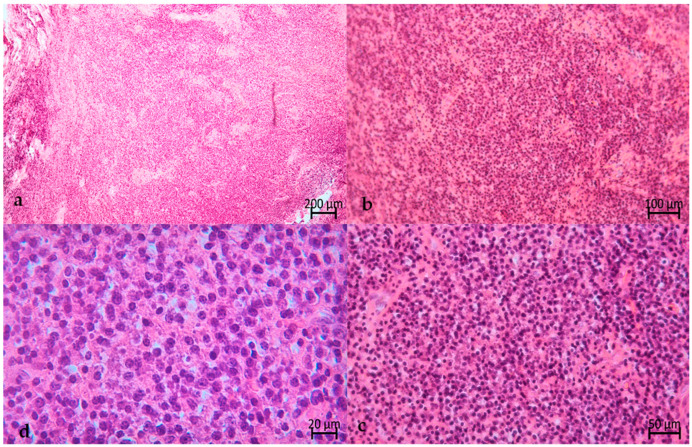
Histological features of the lesion. H&E stain. Primovert ZEISS^®^ (Jena, Germany). (**a**) Image with 4× magnification giving an overview of the lesion, full of plasma cells. (**b**,**c**) Images of 10× and 20× magnification of the central area. (**d**) Image of 40× magnification of the mature plasma cells, which show an oval morphology with eosin cytoplasm, round eccentric nuclei, and perinuclear hof.

The biopsy was immediately fixed in formaldehyde solution and referred to a private laboratory for histological and immunohistochemical evaluation. The hematoxylin and eosin (H&E) stain showed the proliferation of mature plasmacellular elements, which were positive for CD138, and a small infiltrate of B and T lymphocytes, both having small dimensions (Figure 8). In addition, the immunohistochemical assay results for kappa or lambda chains were negative. 

All laboratory tests results were negative; they included blood count, reactive C protein, protein Electrophoresis, total protein, urine, liver function, and renal function (Table 1). 

**Figure 9:**
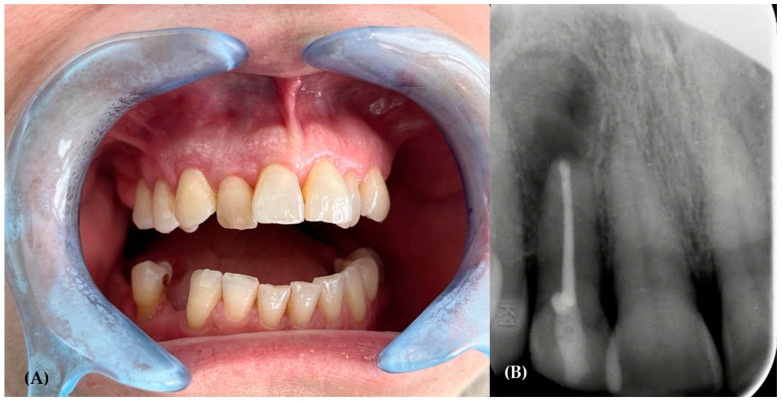
(**A**) Intraoral image after 3 months of healing; tissues appeared normal. (**B**) Endoral radiograph showing the process of bone healing.

A 3-month follow-up showed the presence of normal soft tissues around the upper lateral incisor (Figure 9A), while intraoral radiography (Figure 9B) demonstrated the bone tissue healing process in progress. The patient is currently undergoing treatment by the hematologist. The hematologist did recommend repetition of the blood and urine tests, with particular attention to the kappa and lambda chains and the free light chain (FLC) ratio, and imaging investigation (Magnetic Resonance Imaging—MRI and Positron Emitting Tomography—PET). The patient, however, still has to undergo a blood test and imaging scans to definitively exclude multiple myeloma and decide on the appropriate strategic therapy.

## 3. Discussion

Plasmacytomas originating from bone are an uncommon form of proliferation, representing approximately 3 to 5% of all plasma cell lesions. [5]. They are more prevalent in males, with a male-to-female ratio of 3:1, and typically manifest around the age of 50–60 on average [9]. 

As regards their localization in the bone marrow of the head and neck region, solitary bone plasmacytomas tend to be more prevalent in the body, the ramus, and the angle of the mandible than the maxilla, due to the presence of bone marrow in these areas [10]. 

In this case report, the diagnosis of solitary plasmacytoma in the anterior maxilla, which has still to be confirmed through further analysis, at the level of the upper lateral incisor was an example of a rare localization of this type of lesion in the head region [11], showing the challenge in the diagnosis of this pathology; therefore, plasmacytomas are more common in long bones and vertebrae, while they are rare in the jaw [12]. 

This case study highlights the importance of accurate clinical and radiological examinations, as well as an adequate background in oral medicine and in hematologic diseases with oral manifestation in case of rare manifestations of these diseases. Indeed, dentists can play a key role in the diagnosis of these silent diseases, such as the case of Ali et al. [13]. The authors, in their article, underline the possibility of early diagnosis from dental practitioners in these diseases, which are often asymptomatic in their early stages [13]. 

Histologically, these lesions are usually easily recognizable as being plasmacytic in nature. 

However, the histological features, which rarely present an anaplastic morphology, need immunohistochemical confirmation for differential diagnosis with other hematologic diseases such as plasmablastic lymphoma, plasma cell myeloma, lymphoma, leukemia, Ewing’s sarcoma, and neuroendocrine metastasis [14]. Immunohistochemistry demonstrates positive staining for CD138, CD38, CD79a, IRF4 (MUM1), and CD56 (in 80% of cases). They may exhibit varying degrees of staining for other B cell markers, such as CD19 and CD20. The majority of cases do not stain positive for CD45 and epithelial markers. They demonstrate either kappa or lambda clonality, which can be confirmed through immunohistochemistry or flow cytometry [14]. 

The histological evaluation of the biopsy of this case report confirmed the presence of mature plasmacellular elements characterized by an oval morphology with eosin cytoplasm, round eccentric nuclei, and perinuclear hof; additionally, the presence of small infiltrates of B and T lymphocytes was detectable. The immunohistochemical evaluation demonstrated positivity for CD138, while the immunohistochemical assay for kappa or lambda chains produced negative results.

Clinically, patients experience either painful or painless swelling in the jaw. Additional symptoms may include tooth migration and bleeding. Radiologically, the lesion is described as a well-defined, multiloculated, radiolucent area [3]. 

Solitary plasmacytoma of the jawbone radiologically might appear as an odontogenic tumor or a cystic lesion [15]. In 2017, the International Myeloma Working Group provided guidelines on imaging exams for obtaining a correct diagnosis. In particular, PET/CT scan is considered essential for the diagnosis of solitary plasmacytoma of the bone [15]. 

Later, in 2019, the same group also underlined the importance of MRI in assessing and characterizing the dimension of the local tumor and eventually localizing other lesions in the whole body [16]. In this case, the patient had the clinical sign of swelling in the maxilla localized at the level of the right upper lateral incisor, which is not a specific sign of the solitary plasmacytoma, so a differential diagnosis with periapical granuloma was required. For obtaining a correct and definitive diagnosis, blood and urine tests play a crucial role. Indeed, there should be no presence of M-protein in the serum and urine as well as an absence of hypercalcemia, thrombocytopenia, neutropenia, and renal failure. 

In addition, blood tests might indicate a prognosis such as an abnormal serum free light chain ratio, which is reported as a risk factor for progression to multiple myeloma [17]. 

While there may be a presence of monoclonal paraproteins in the urine or serum, other clinical and radiological indications of multiple myeloma should be absent, such as hypercalcemia, kidney dysfunction, anemia, more than 10% clonal plasma cells in the bone marrow, and multiple bone lesions visible on imaging [2]. 

In this case, the results of the early analysis of the patient’s blood were not positive for the above-mentioned parameters.

Usually, primary differential diagnoses include multiple myeloma and extraosseous plasmacytoma. Clinically and radiologically, solitary bone plasmacytoma can be distinguished from multiple myeloma based on these aspects. Extraosseous plasmacytomas occurring in tissues other than bone are extremely rare (less than 1% of plasmacytic lesions). For example, Abe et al. [18] found an extramedullary plasmacytoma in the sublingual gland. Extraosseous plasmacytomas tend to affect the upper respiratory tract and cervical lymph nodes, but share similar characteristics and immunohistochemical profiles with their bone counterparts [19]. 

The treatment of solitary plasmacytoma of the jaw may consist of different modalities, but surgical intervention plays a crucial role in the management of solitary plasmacytoma of the jaw. Particularly in cases where the tumor causes significant structural damage or compromises oral function, surgery should consider the functional and cosmetic aspects of jaw reconstruction and achieve complete resection of the tumor. 

Depending on the extent of the disease, various surgical techniques, such as segmental resection, curettage, or enucleation, may be employed. Adjuvant radiation, involving the use of high-energy X-rays or other radiation sources, is often recommended following surgery to minimize the risk of local recurrence [20]. 

Finally, systemic chemotherapy is typically reserved for cases of solitary plasmacytoma of the jaw that exhibit aggressive behavior or have progressed to multiple myeloma; chemotherapy may be administered before surgery to shrink the tumor, or after surgery as an adjuvant to eliminate residual cancer cells [21]. 

In recent years, several novel treatment approaches have emerged for the management of solitary plasmacytoma of the jaw; these include targeted therapies, immunomodulatory agents, and monoclonal antibodies. Targeted therapies, such as proteasome inhibitors, specifically inhibit key molecular pathways involved in the growth and survival of plasma cells. Immunomodulatory agents enhance the immune response against cancer cells. Monoclonal antibodies can selectively target plasma cells, leading to their destruction [22].

In this case report, as the lesion did not cause important structural damage, but only involved the apical part of the root of the right upper lateral incisor, surgical intervention was performed involving enucleation of the lesion associated with root canal treatment and apicoectomy of the right upper lateral incisor. The patient is currently undergoing treatment by a hematologist.

## Figures and Tables

**Table 1:** Overview of the laboratory tests, which did not show any evidence of current systemic involvement of the above-described plasmacytoma localized in the maxillary jaw.

Laboratory Test	Resulting Value	Unit of Measurement	Reference Value
**White blood cell: total leucocytes** (hydrodynamic focusing and flow cytometry) Neutrophils Lymphocytes Monocytes Eosinophils Basophils	6.10 47.8 43.1 7.4 1.5 0.2	10^3^/μL % % % % %	4.00–10.00 10^3^/μL 40.0–68.0% 20.0–45.0% 2.0–10.0% 0.5–5.0% 0.0–2.0%
**Erythrocyte count**	4.35	10^6^/μL	4.00–5.10 10^6^/μL
**Platelet count**	161	10^3^/μL	130–450 10^3^/μL
**Total plasma protein values** (protein electrophoresis) Albumin Alpha 1 globulin Alpha 2 globulin Beta globulin Gamma globulin	6.89 59.1 2.1 10.2 10.6 18.0	% % % % % %	5.20–8.50% 52.0–68.0% 1.4–4.5% 6.5–13.5% 8.0–15.0% 10.5–20.5%
**C-reactive protein (CRP)**	0.70	mg/L	Up to 5.0 mg/L
**Erythrocyte sedimentation** **rate (ESR)** (immunoturbidimetric method)	20	mm/h	<15 mm/h
**Protein in urine**	0.00	mg/dL	0–10 mg/dL
**Kappa free light chains** **Lambda free light chains** **Ratio of kappa/lambda**	4.33 6.7 0.63	mg/L mg/L	3.3 to 19.4 mg/L 5.71 to 26.3 mg/L 0.26 to 1.65

## Data Availability

Data will be made available by the corresponding author upon reasonable request.
